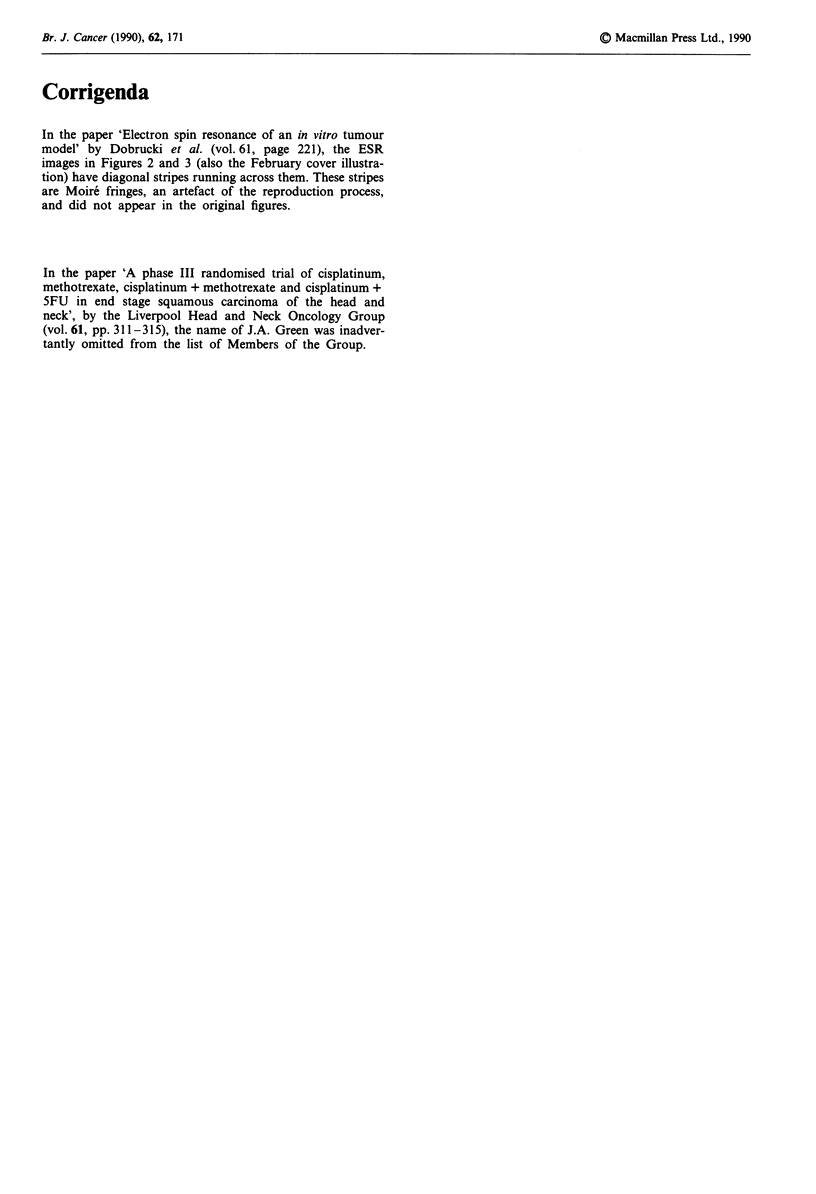# Corrigenda

**Published:** 1990-07

**Authors:** 


 © Macmillan Press Ltd., 1990

### Corrigenda

In the paper 'Electron spin resonance of an *in vitro* tumour model' by Dobrucki *et al.* (vol. 61, page 221), the ESR images in Figures 2 and 3 (also the February cover illustration) have diagonal stripes running across them. These stripes are Moiré fringes, an artefact of the reproduction process, and did not appear in the original figures.

In the paper 'A phase III randomised trial of cisplatinum, methotrexate, cisplatinum + methotrexate and cisplatinum + 5FU in end stage squamous carcinoma of the head and neck', by the Liverpool Head and Neck Oncology Group (vol. **61**, pp. 311–315), the name of J.A. Green was inadvertantly omitted from the list of Members of the Group.